# Impact of Medium-Sized Extracellular Vesicles on the Transduction Efficiency of Adeno-Associated Viruses in Neuronal and Primary Astrocyte Cell Cultures

**DOI:** 10.3390/ijms22084221

**Published:** 2021-04-19

**Authors:** Orsolya Tünde Kovács, Eszter Soltész-Katona, Nikolett Marton, Eszter Baricza, László Hunyady, Gábor Turu, György Nagy

**Affiliations:** 1 Department of Genetics, Cell- and Immunobiology, Semmelweis University, Nagyvárad tér 4, 1089 Budapest, Hungary; kovacs.orsolya1@med.semmelweis-univ.hu (O.T.K.); baricza.eszter@med.semmelweis-univ.hu (E.B.); 2 Department of Physiology, Semmelweis University, Tűzoltó street 37-47, 1094 Budapest, Hungary; soltesz-katona.eszter@med.semmelweis-univ.hu (E.S.-K.); hunyady.laszlo@med.semmelweis-univ.hu (L.H.); 3 Jahn Ferenc Dél-pesti Hospital, Department of Radiology, Köves street 1, 1204 Budapest, Hungary; marton.nikol@gmail.com; 4 MTA-SE Laboratory of Molecular Physiology, Eötvös Loránd Research Network, 1085 Budapest, Hungary; 5 Department of Rheumatology & Clinical Immunology, Semmelweis University, Árpád fejedelem street 7, 1023 Budapest, Hungary

**Keywords:** adeno-associated virus, AAV, extracellular vesicles, microvesicles, N2A, primary astrocyte cells

## Abstract

(1) Adeno-associated viruses (AAV) are safe and efficient gene therapy vectors with promising results in the treatment of several diseases. Extracellular vesicles (EV) are phospholipid bilayer-surrounded structures carrying several types of lipids, proteins, and nucleic acids with the ability to cross biological barriers. EV-associated AAVs might serve as new and efficient gene therapy vectors considering that they carry the benefits of both AAVs and EVs. (2) We tested vesicle-associated AAVs and vesicles mixed with AAVs on two major cell types of the central nervous system: a neural cell line (N2A) and primary astrocyte cells. (3) In contrast to previously published in vivo observations, the extracellular vesicle packaging did not improve but, in the case of primary astrocyte cells, even inhibited the infection capacity of the AAV particles. The observed effect was not due to the inhibitory effects of the vesicles themselves, since mixing the AAVs with extracellular vesicles did not change the effectiveness. (4) Our results suggest that improvement of the in vivo efficacy of the EV-associated AAV particles is not due to the enhanced interaction between the AAV and the target cells, but most likely to the improved delivery of the AAVs through tissue barriers and to the shielding of AAVs from neutralizing antibodies.

## 1. Introduction

Adeno-associated virus (AAV) is one of the smallest viruses belonging to the Parvovirus family with a single-strand DNA genome. In the past few years, AAVs have become emerging vectors for gene therapy or vaccine development vectors [1] as a result of numerous clinical trials with favorable outcomes for the treatment of several inherited genetic diseases including those of the eye [2,3], of the central nervous system (CNS) [4,5,6,7,8], hemophilia A and B [9,10], spinal muscular atrophy [11], and lipoprotein lipase deficiency [12]. Most gene therapies are virus-based techniques, which aim to transfer nucleic acid sequences to target cells. AAV particles have excellent safety and efficacy profiles; they are non-pathogenic, have a low immunogenic profile, and show long-term episomal expression. However, there are some major limitations of the AAV gene transfer vectors as well. These include the limited genome-packaging capacity, which is only ∼5 kb [13], and up to two-thirds of the human population is seropositive to AAV [14] resulting in preexisting neutralizing antibodies in the human sera [15,16,17,18]. It has been discovered recently that specific AAV receptors expressed by susceptible cells are essential for the transduction of multiple serotypes of AAV particles, as they interact with AAV capsid ligands [19].

Gene transfer might be limited by biological barriers (e.g., plasma membranes) in some target tissues or organ-specific barriers. This is especially relevant to certain preferred gene therapy administration routes (e.g., intravitreal injection), influencing the viral uptake by cells [20,21,22]. Therefore, emerging new therapeutic vectors are essential for developing novel gene delivery approaches.

Extracellular vesicles (EV) are phospholipid bilayer enclosed structures, which are actively released by all cells in nature. EVs play an important role in cell-to-cell communication by carrying proteins, lipids, or nucleic acids including RNAs [23], microRNAs [24,25], other small non-coding RNAs, and DNAs [26]. In addition to their physiological roles, EVs may also play an important role in several pathological conditions [27]. Therefore, they have attracted increasing attention as biomarkers [28,29,30,31] in the past few years. Moreover, EVs are also promising therapeutic tools. In gene therapy, targeting may be especially challenging. Due to their capacity to cross lipid membrane barriers, EVs may serve as carriers of viruses. Because of the heterogeneity of size [32,33], biogenesis [34], and cargo [35,36,37] of vesicles, standardization of isolation, purification, and characterization of extracellular vesicles require special efforts from the scientific community for the development of vesicle-mediated therapies [38,39,40,41].

It has already been described that exosomes (small EVs of multivesicular body origin) may be associated with several types of viruses including HIV-1 [42], picornavirus [43], HBV [44], and adenovirus [45]. Maguire et al. reported that AAV capsids were associated with EVs and observed by using transmission electron microscopy that some vesicles contained AAVs as an internal cargo as well [46]. Exosome-associated AAV vectors (referred to as “vexosomes” or exo-AAV) have been demonstrated to mediate higher transduction efficiencies in vivo as compared to standard AAV vectors in the retina [47], the inner ear [48], the liver [49], and the CNS [50] in mice. Furthermore, it has been shown that exosomes can shield AAV capsids from neutralizing antibodies, and it is non-immunogenic in mice [49,51].

It has been also demonstrated that the vesicular membrane protects the AAVs from the preexisting antibodies. Here, we set to address the question of whether the AAV-EV association results in enhanced uptake of the AAV cargo by cells of the CNS. Given that current gene therapeutic approaches often target the CNS, here, we focused on the uptake of the AAV-EV complexes by murine neurons and primary astrocyte cells. We chose the AAV DJ vector because it has high efficacy to infect neurons and astrocytes [52].

To this end, we carried out a quantitative assessment of the efficacy of the AAV-assisted gene delivery in vitro when packaged into EVs. We tested the transduction efficiency of (i) AAV, (ii) medium-sized EV-associated AAV (mEV-AAV), and (iii) the mixture of mEV with AAV (mEV + AAV) in vitro.

Our present data suggest that EV packaging does not improve the infection capacity of the AAV particles. In the case of the primary astrocyte cells, it even reduced the cellular uptake of the virus particles as compared to AAV. The observed effect was not due to an inhibitory effect of the vesicles, since mixing AAVs with EVs did not influence the cellular uptake.

## 2. Results

### 2.1. mEV-AAV Complexes Can Transduce Primary Astrocytes and N2A Cells In Vitro

**Figure 1:**
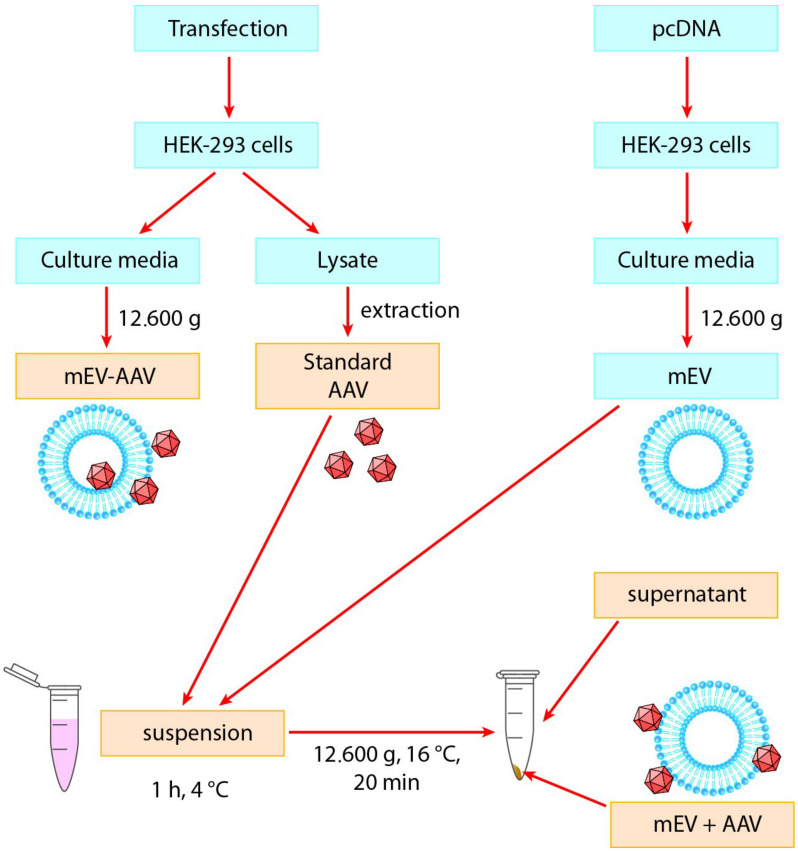
Experimental design of this study. Standard “AAV”, “mEV-AAV”, mEV and AAV “suspension”, “mEV + AAV”, and “supernatant” preparations were added to neuronal and primary astrocyte cell cultures (indicated with yellow). AAV: Adeno-associated virus, mEV-AAV: medium-sized extracellular vesicle-associated AAV, mEV + AAV: mixed mEV with AAV.

To study the impact of mEV on the transduction efficiency of AAV in vitro, we used the experimental system shown in Figure 1. We isolated AAVs from HEK-293 cells and separated EVs from the conditioned medium of HEK-293 cells.

To objectively compare the transduction efficiency of the EV-associated AAVs with native AAVs, we used standard “AAV” and “mEV-AAV” as described previously [47]. Furthermore, we also prepared a “suspension” (a simple mixture of mEVs and AAVs at 4 °C) and collected the pellet (“mEV + AAV”) and the “supernatant” after a centrifugation step. Based on our previous observations, a large amount of mEVs can be isolated from the conditioned medium of untransfected cells. Therefore, we aimed to examine the transduction efficiency, by using vesicles derived from non-AAV-producing cells and mixed with isolated AAVs.

Standard “AAV”, “mEV-AAV” (released by AAV-producing cells), the “suspension” (a mixture of AAV and mEV), the “mEV + AAV” pellet, and the “supernatant” samples were freshly prepared for each experiment and added to mouse primary astrocytes or N2A cell cultures in vitro (Figure 1). For each sample, the AAV titer was determined by quantitative PCR.

Next, we transduced cultured primary astrocytes and N2A cells, which were plated 24 h (primary astrocytes) or 48 h (N2A cells) earlier on poly-d-lysine-coated glass coverslips with freshly prepared AAV and mEV combinations or complexes (as described above). The AAV vectors carried the cDNA of the mVenus fluorescent protein. We used confocal microscopy to detect the fluorescence 5 days (N2A cells) or 10 days (primary astrocytes) after the exposure of the cells to the AAV and mEV complexes or combinations.

**Figure 2:**
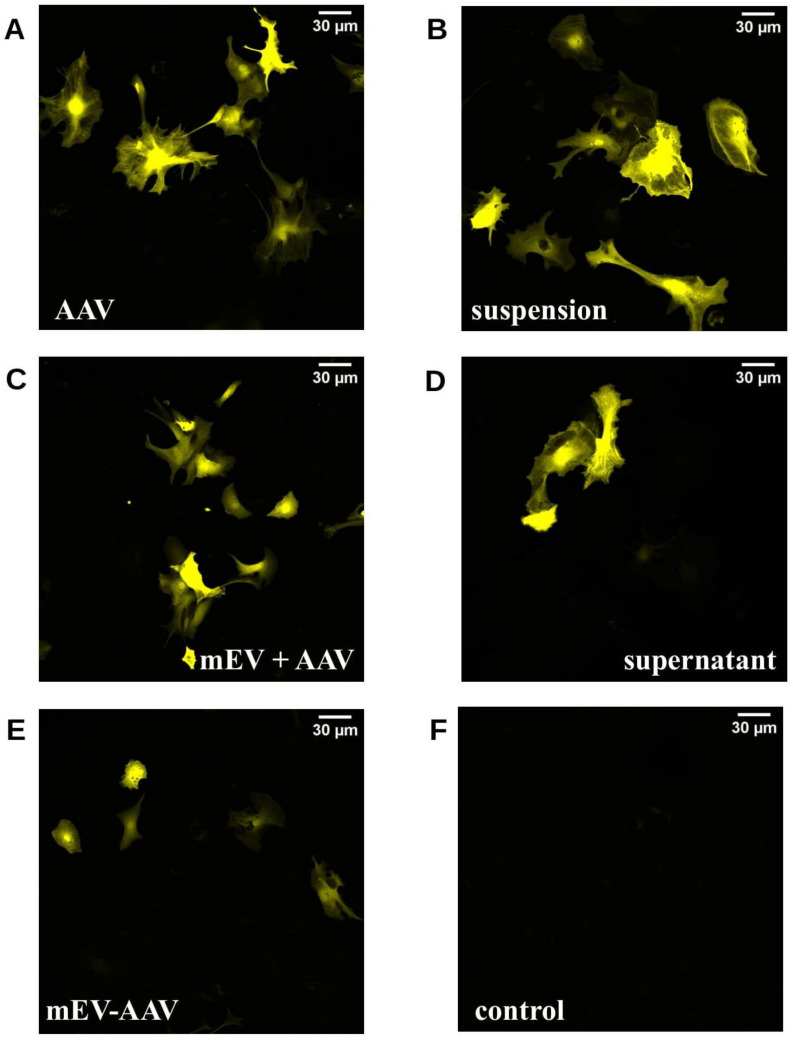
Confocal microscopic images of mouse astrocyte primary cell cultures 10 d after treatment. Astrocytes were transduced in vitro with different AAV treatments containing freshly prepared AAV and mEV combinations or complexes. (**A**) standard “AAV”, (**B**) “suspension” (a mixture of AAV and mEV), (**C**) “mEV + AAV” (co-centrifugation pellet of the previous suspension), (**D**) supernatant, (**E**) mEV-AAV (mEV-associated AAV released by AAV producing cells), (**F**) negative control.

Figure 2 shows representative confocal images of all applied preparations in mouse astrocytes.

### 2.2. mEV-AAV Impairs Transduction Efficacy In Vitro in Primary Astrocyte Cells

**Figure 3:**
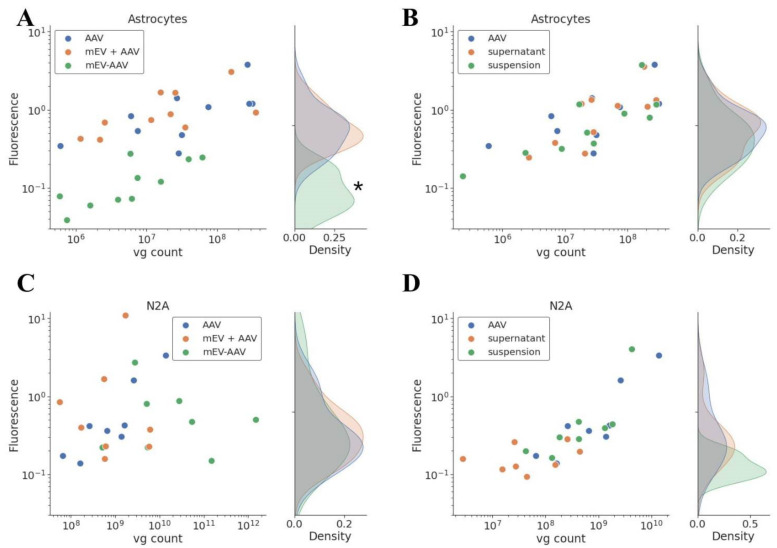
Quantitative analysis of transgene expression. Measured fluorescence was represented with applied vg. EV-packaging reduced the infection capacity of the AAV particles in primary astrocyte cells. Mixing the AAV with EV did not influence the effectiveness. (**A**) Primary astrocytes, “AAV”, “mEV + AAV”, and “mEV-AAV” treatment, *n* = 5, * indicates significant difference between “mEV-AAV” and “AAV” transduction efficiency, *p* < 0.0001. (**B**) Primary astrocytes, “AAV”, “supernatant”, and “suspension” treatment, *n* = 5. (**C**) N2A cell culture, “AAV”, “mEV + AAV”, and “mEV-AAV” treatment, *n* = 4. (**D**) N2A cell culture. “AAV”, “supernatant”, and “suspension” treatment, *n* = 4.

To quantify the transduction efficacy, we carried out the same experiment on poly-d-lysine-coated 96-well plates and measured the fluorescence of the cells in Varioscan spectrophotometer. Experiments were performed in triplicates to minimize random error and experimental bias. The fluorescence was detected 5 (N2A cells) or 10 (primary astrocytes) days after exposure to standard “AAV”, “mEV-AAV”, mEV and AAV “suspension”, “mEV + AAV”, and “supernatant”, and the results were plotted against the number of applied viral genomes (vg) on Figure 3.

As shown in the figure, the transduction efficacy of “mEV + AAV”, mEV and AAV “suspension” and the “supernatant” was similar to that obtained in the case of the standard “AAV” in the case of both cell lines. Surprisingly, the “mEV-AAV” complexes did not improve but rather decreased the transduction efficiency in primary astrocyte cells (*p* < 0.0001, multiple linear regression model). In N2A cells, however, the efficiency was similar to the “AAV” efficiency.

Multiple linear regression analysis indicated that the measured fluorescence increased substantially in Figure 3A,B (*p* < 0.0001) and D (*p* < 0.05) with the number of applied vg as determined by quantitative PCR.

## 3. Discussion

**Figure 4:**
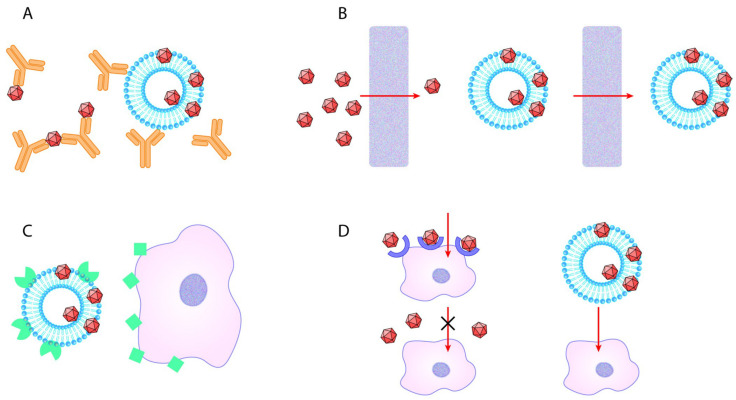
Potential advantages of EV-AAV over standard AAV. (**A**) EV-AAV can shield AAV capsids from humoral immune response. (**B**) EV-AAV may traverse in vivo biological barriers, while transferring AAV either attached to them or inside of them more effectively compared to standard AAV. (**C**) By producing EV with targeting proteins of their surface, gene transfer may be easily carried out. (**D**) AAV particles are not taken up by cells, which do not express AAV-receptors. However, vesicles may enter cells through multiple receptors, thus making previously non-susceptible cells to be able to uptake AAV transferred by EV.

Vesicle-bound AAVs are recently described as promising therapeutic vectors with considerable potential to deliver genetic material into target cells [50,53]. The EV-AAV approach proved to be beneficial in vivo in experimental model systems by protecting the viral vector by the membrane of the EV from neutralizing antibodies. Here, we addressed the question of cellular uptake by the two major cell types of the CNS. Figure 4 summarizes the potential benefits of the EV-AAV gene delivery system [54,55,56,57,58,59,60,61]. In the in vitro system, there were no tissue barriers; therefore, we could explore the capacity of the EVs to selectively enhance the cellular uptake of the AAVs. Surprisingly, in this in vitro system, the “mEV-AAV”, released by AAV-producing cells either did not affect or decreased the infection capacity of the AAV particles. Furthermore, if the AAV particles were mixed with EVs originating from naïve HEK-293 cells, the transduction efficiency of the infection was not affected. These results are somewhat surprising, since several reports describe the higher transduction efficiency of exo-AAV compared to standard AAV in vivo [46,47,48,49,50,51,62,63]. We used differentiating N2A cells, representing neural cells, and primary astrocytes, representing glial cells. We chose these two cell types because EV-mediated AAV gene delivery is considered a highly promising neuroscience tool.

One possible explanation of our findings is that the transduction with EVs might need special receptor expressions on the cell surfaces, and these two cell types do not express these receptors. However, this seems unlikely, since based on the in vivo experiments, there was no significant preference to one or other type of cells. As a second possibility, the infection was inhibited because AAV particles were hidden inside the vesicles and were not as readily available to the surface AAV receptors of the target cells. This would also suggest that the in vivo observed infection-augmenting effects of the EV-associated AAV particles were not particularly due to the enhanced interaction between AAV particles and target cells, but most likely due to the improved delivery of AAV through tissue barriers, and/or because of protecting effects of vesicles against neutralizing antibodies, as described previously [48]. The same report also described that mEV-AAV had a higher transduction rate compared to standard AAV in vitro on primary murine neurons, SH-SY5Y cells, melanocytes, and HeLa cells; however, the AAV titers in these experiments are not clear [48]. A third possible explanation is that, as far as we know, only AAV1/AAV2/AAV5/AAV6/AAV8/AAV9 serotypes were used for EV-AAV preparation for the published in vivo studies. Here, we used the AAV-DJ serotype, which might already have an enhanced infection rate compared to the commonly used AAV1 or AAV2 viruses. Thus, the transduction efficiency, in this case, might not be further enhanced in vitro. Moreover, it was reported that the EV-AAV association also showed some serotype specificity, as there were significantly more AAV1 associated with EVs than AAV2 [46]. This result suggests that the amount of the packaged AAV into EVs was serotype-dependent, and thus, the effect of the packaging could be well dependent on the particular AAV used. One further explanation might be that in our experiments, we used mEVs instead of the exosomes. We harvested mEV-AAV from the pellet obtained by centrifuging at 12.600× *g*. We chose this spin force vs. higher speed, as it minimizes co-pelleting of free AAV from the media [46]. The other advantage of the mEVs over exosomes is the easier isolation procedure with equipment readily available to researchers in usual cell biology laboratories. The mEV fraction has been already shown to enhance the transduction efficiency [51]. We also tried to mix isolated AAVs with purified EVs, since it has been shown that AAVs are not only packaged into the interior of EVs but also are attached to the surface of them [46], raising the possibility that the vesicle surface association with AAV might be advantageous during the transduction of the cells. According to our results, the AAV-EV mix did not show higher efficiency compared to standard AAV. Additionally, there was no enrichment in AAV in the vesicle fraction after centrifugation in these mixed samples, as determined with titer measurements in the pellet and the supernatant, and there was no enhancement in the effectiveness with these samples.

The small number of cell lines is a limitation in our study. We selected the two cell lines, which represent major cell types in the brain, neurons, and astrocytes; however, as discussed above, the presence or absence of specific receptors on the cell surface may influence the effectiveness of the different AAV delivery methods.

## 4. Materials and Methods

### 4.1. Cell Culture

The human AAV-293 cell line (HEK-293 cell line optimized for the packaging of AAV virions) was obtained from Agilent (240073). The mouse Neuro-2A (N2A) cell line (CCL-131) was obtained from American Type Culture Collection (ATCC, Manassas, VA, USA). Both cell lines were cultured in high glucose Dulbecco’s modified Eagle’s medium, supplemented with 10% fetal bovine serum (FBS), and 100 U/mL penicillin, 100 μg/mL streptomycin (Invitrogen, Waltham, MA, USA) in a humidified atmosphere supplemented with 5% CO2 at 37 °C.

Mouse astrocyte primary cell cultures were prepared from postnatal day 1 of the C57BL/6 mouse brain. After decapitation, the brain of P0–1 pups was taken out and the cerebellum, and the thalamus were removed on ice in a dissociation medium (DM for 50 mL: 45 mL HBSS, 670 μL 45% Glucose (2.5 M stock), 5 mL kynurenic acid (10 mM), 500 μL MgCl2 (100 mM)). We removed the pia mater completely and transferred the hemispheres into a new tube. We chopped the brain into small pieces using a scalpel and washed twice with DMEM. Then, we pipetted up and down 8–10× with a 5 mL pipette until the tissue was completely dissociated into individual cells. Cells were resuspended in DMEM and plated to a 10 cm diameter Petri dish in 10 mL. Next day, the medium was changed. In 7–10 days, when the confluency was 60–80%, the cells were subcultured into new Petri dishes with the standard Trypsin-EDTA protocol.

For fluorescence measurement and confocal microscopy, cells were plated the day before the treatment was carried out on adherent cells.

### 4.2. Plasmid Construction

Vector XX680 was obtained from the University of Pennsylvania (Philadelphia, PA, USA). We replaced the existing promoter in pAAV-EF1a-DIO vector with an astrocyte-specific promoter (glial fibrillary acidic protein, GFAP). GFAP was derived from an Addgene plasmid hGFAP-NLS-HA-Dre-P2A-BFP [64]. We replaced the promoter after PCR amplification of the GFAP and digestion of it with MluI/BamHI restriction enzymes. mVenus was inserted between BamHI/EcoRI sites. Constructs were packaged into adeno-associated virus pseudotype DJ (AAV-DJ) (Cellbiolabs, San Diego, CA, USA) using the triple-transfection technique, as previously described [65]. Titers of each virus were set to be between 1 × 10^7^–1 × 10^8^ vector genomes/mL by qPCR using Takara AAVpro^®^ Titration kit (Takara Bio Inc., Kusatsu, Shiga Prefecture, Japan).

### 4.3. Preparation of EV-Depleted FBS

EV-depleted FBS was prepared to avoid the presence of FBS-derived EV in EV preparations. FBS (Invitrogen) was ultracentrifuged by a 120.000× *g* for 16 h at 4 °C in a type MLA-55 rotor (Optima Max-XP, Beckman Coulter, Brea, CA, USA). The supernatant was aspirated cautiously and transferred to a new tube and then filtered through 0.22 μm filter (Millipore, Burlington, MA, USA) and was stored at −20 °C until use.

### 4.4. AAV Vector Production and Medium-Sized Extracellular Vesicle (mEV) Isolation

AAV vectors and mEV-AAVs were produced in human HEK-293 cells. Briefly, a triple transfection with GFAP-mVenus, XX680, and AAV-DJ plasmids was performed using the calcium phosphate method in two 15 cm Petri dishes as described previously [65]. Plasmid DNA was added in water with 2.5 M CaCl2 (final concentration: 125 mM) and 2x Hepes-buffered solution (HBS) (42 mM Hepes, 15 mM dextrose/D-glucose, 1.4 mM Na2HPO4x7H2O, 10 mM KCl, 274 mM NaCl at pH 7.1). Two control 15 cm Petri dishes were transfected with plasmid cloning DNA to exclude the impact of transfection on EV formation. 

Six to eight hours after the transfection, the culture medium of both the triple transfected and control HEK-293 cells was changed to EV-depleted FBS-containing medium to avoid the separation of FBS-derived EV in the EV preparations.

At 48 h post-transfection, standard AAV vectors were extracted from the lysates of triple transfected cells by using Takara AAVpro^®^ Extraction Solution kit (Takara Bio Inc., Kusatsu, Shiga Prefecture, Japan). Then, AAV vectors were concentrated using an Amicon^®^ Ultra-15 centrifugal device with 100 kDa molecular weight cutoff (Millipore) and PBS (this preparation was referred to as “AAV”).

The harvested conditioned medium was used for EV separation by differential centrifugation and filtration steps. First, cells and apoptotic bodies were removed by sequential, 10 min 300 g and 20 min 2.000 g centrifugations, respectively, using an Eppendorf Centrifuge 5702 R. The supernatant was transferred to a fresh tube and then filtered sequentially by gravitation through 5 and 0.8 μm filters (Millipore) to remove large-sized vesicles (lEV). The filtrate was centrifuged at 12.600× *g* for 20 min (Eppendorf Centrifuge 5427 R). The medium-sized vesicle (mEV) pellet was resuspended in PBS (resulting in the “mEV-AAV” and the mEV preparations from the AAV-transduced and control cells, respectively). Extracted and concentrated “AAV” was mixed with mEV samples derived from the conditioned medium of the control HEK-293 cells and stored for 1 h at 4 °C (“suspension” of AAV and mEV). After centrifugation at 12.600× *g* for 20 min, the pellet (“mEV + AAV”) and the supernatant (“supernatant”) were added to mouse astrocyte primary cell culture or N2A cells similarly to “AAV”, “mEV-AAV”, and “suspension” preparations. All samples used for in vitro treatments were prepared freshly for each experiment (Figure 1).

### 4.5. Vector Quantification

To obtain the titer of “AAV”, “mEV-AAV”, “suspension”, “mEV + AAV”, and “supernatant” samples, a quantitative TaqMan PCR was carried out by using AAVpro^®^ Titration Kit Ver. 2 (Takara). The quantification was based on the amplification of inverted terminal repeats of AAV. First, the free AAV genomic and plasmid DNAs derived from the host cells were digested with DNAse I at 37 °C. Next, DNAse I was inactivated for 10 min at 95 °C. Then, we applied PCR using SYBR Premix Ex Taq II. A standard curve was prepared using serial dilutions of an AAV plasmid of a known molar concentration. The quantitative PCR was performed in a LightCycler^®^ 480 Real-Time PCR System (Roche Life Science, Penzberg, Germany) using the following conditions: 1 cycle, 95 °C 2 min; 35 cycles 95 °C 5 s, 60 °C 30 s.

### 4.6. Confocal Laser-Scanning Microscopy

To obtain confocal microscopy images, primary mouse astrocytes and N2A cells were plated on poly-d-lysine-coated glass coverslips. Ten days (astrocytes) or 5 days (N2A cells) after exposure to the different preparations, the medium was changed to modified Kreb’s-Ringer medium, and fluorescent protein expression was examined in living cells at 37 °C with Zeiss LSM 710 confocal laser-scanning microscope (Zeiss, Oberkochen, Germany) using an EC Plan-Neofluar 20×/0.50 M27 objective.

### 4.7. Fluorescence Measurement, Data Analysis

mVenus expression and fluorescence intensities were determined by exciting at 510 nm and measuring emission at 535 nm, respectively, using a Thermo Scientific Varioskan Flash multimode plate reader (Thermo Scientific, Waltham, MA, USA) 10 days (primary astrocytes) or 5 days (N2A cells) after treatment of the cell cultures.

### 4.8. Statistical Analysis

Statistical analysis was carried out using the software GraphPad Prism version 9.0.0 (San Diego, CA, USA). We fitted a multiple linear regression model Measured Fluorescence ~ Vector Genome + Treatment, where the Treatment factor represents the used AAV treatments (“AAV”, “mEV-AAV”, “suspension”, “mEV + AAV”, “supernatant”). Measured Fluorescence and Vector Genome values were log-transformed prior to the statistical analysis. We report the *p*-values of the multiple linear regression model coefficients (using “AAV” treatment as the reference level); *p* < 0.05 was considered as a significance threshold. To test the effect of treatments, we also compared the statistical model with a baseline model, Measured Fluorescence ~ Vector Genome. 

## 5. Conclusions

In conclusion, EV-assisted AAV treatment does not have a general enhancing effect in vitro, and the in vivo observed effect might be the result of neutralizing antibody shielding effect and the ability of the EVs to pass over physical and biochemical barriers. Further objective examinations are needed to study the effectiveness of the AAV-assisted gene delivery when packaged into medium-sized extracellular vesicles.
